# Valorization of Chicken Slaughterhouse Byproducts to Obtain Antihypertensive Peptides

**DOI:** 10.3390/nu15020457

**Published:** 2023-01-15

**Authors:** Francisca Isabel Bravo, Enrique Calvo, Rafael A. López-Villalba, Cristina Torres-Fuentes, Begoña Muguerza, Almudena García-Ruiz, Diego Morales

**Affiliations:** Nutrigenomics Research Group, Department of Biochemistry and Biotechnology, Universitat Rovira i Virgili, 43007 Tarragona, Spain

**Keywords:** angiotensin-converting enzyme-inhibitory activity, blood pressure, hydrolysates, hypertension, endothelial dysfunction

## Abstract

Hypertension (HTN) is the leading cause of premature deaths worldwide and the main preventable risk factor for cardiovascular diseases. Therefore, there is a current need for new therapeutics to manage this condition. In this regard, protein hydrolysates containing antihypertensive bioactive peptides are of increasing interest. Thus, agri-food industry byproducts have emerged as a valuable source to obtain these hydrolysates as they are rich in proteins and inexpensive. Among these, byproducts from animal origin stand out as they are abundantly generated worldwide. Hence, this review is focused on evaluating the potential role of chicken slaughterhouse byproducts as a source of peptides for managing HTN. Several of these byproducts such as blood, bones, skins, and especially, chicken feet have been used to obtain protein hydrolysates with angiotensin-converting enzyme (ACE)-inhibitory activity and blood pressure-lowering effects. An increase in levels of endogenous antioxidant compounds, a reduction in ACE activity, and an improvement of HTN-associated endothelial dysfunction were the mechanisms underlying their effects. However, most of these studies were carried out in animal models, and further clinical studies are needed in order to confirm these antihypertensive properties. This would increase the value of these byproducts, contributing to the circular economy model of slaughterhouses.

## 1. Introduction

Hypertension (HTN) or high blood pressure (BP) is a severe medical condition suffered by over 1.28 billion people aged 30–79 years [1]. This chronic disease is also one of the main risk factors for cardiovascular diseases (CVD), as well as brain, heart, or kidney diseases, among others. Consequently, a reduction in its growing incidence is of great importance, targeting its reduction by 33% by 2030 [1]. Hence, there is a need for new strategies to manage HTN [2]. In this context, bioactive peptides, which are small specific protein fragments (2–20 amino-acid residues), released from the native protein via chemical or enzymatic hydrolysis, bacterial fermentation, or food processing, are of increasing interest. Thus, these functional peptides present interesting biological activities such as antioxidant, anti-inflammatory, antidiabetic, and antimicrobial [3]. Regarding HTN, there is strong evidence that they can help to prevent or ameliorate the onset or progression of this disease; at the same time, they might overcome some of the side or adverse effects of existing therapeutic treatments [4,5,6].

Among the diversity of potential dietary sources used to obtain bioactive peptides, agri-food industry byproducts have emerged as an excellent option [5,7]. In particular, those from animal origin are of special interest as they are both rich in proteins [8,9,10,11], which usually can be easily extracted, and inexpensive. In this regard, poultry slaughterhouse byproducts such as blood, feathers, soft meat, skins, or bones, mainly those generated from broilers, stand out due to their elevated generation [12]. This growing industry represented 40% of global meat production in 2020 [13]. Consequently, the management of these wastes must be improved to tackle this growing problem. Thus, the use of these products to obtain bioactive peptides would be an excellent opportunity to valorize these byproducts within the principles of the circular economy [14].

Chicken byproducts are mainly composed of collagen (abundant in bones, legs, feet, etc.) or other structural proteins such as keratin in the case of feathers [15,16]. It has been reported that collagen, from different origins, is an excellent source of peptides with a wide range of bioactivities including anti-skin aging, lipid-lowering, metal-chelating, antioxidant, antidiabetic, or immune modulation [17,18,19]. Moreover, collagen hydrolysates have demonstrated to exert both angiotensin-converting enzyme-inhibitory (ACEi) and antihypertensive activities [18,20,21]. ACE inhibition is a usual and effective methodology to search for antihypertensive peptides [5,22,23] as this enzyme plays an important role in BP regulation [24]. In fact, ACE inhibitors are first-line treatments for HTN as they effectively decrease BP [25]. 

Considering all these facts, the present review is focused on evaluating the potential role of broiler slaughterhouse byproducts as a source of peptides in the management of HTN.

## 2. Materials and Methods

Studies focused on chicken byproducts, peptides and the evaluation of their anti-hypertensive effects were searched using the database Web of Knowledge (https://www.webofscience.com/ (accessed on 8 July 2022). Combinations of several search terms such as “ACE inhibitory activity”, “antihypertensive activity/effect”, “blood”, “blood pressure”, “bone/s”, “byproduct/s“, “chicken”, “feather/s”, “foot/feet”, “leg/s”, “hydrolysate/s”, “liver”, “peptides”, “renin activity”, “residues”, “skin”, “trachea”, “viscera”, “waste/s” were applied. After the search, studies were reviewed, selected and classified according to the studied chicken byproduct, in vitro study and/or antihypertensive effect. 

## 3. Results

### 3.1. Poultry Meat Industry and Chicken Byproducts

Despite the growing vegan, vegetarian, and environmental movements that encourage the consumption of a plant-based diet, the consumption of poultry meat has increased in the last decades. It is estimated that poultry farming will be the livestock sector with the highest projection until 2050 [12,26,27]. According to Food and Agriculture Organization of the United Nations, this industry represented 40% of global meat production in 2020, with chicken the majority poultry species contributing to meat production (90%) [13]. In 2021, the chicken meat production worldwide was estimated at about 80.2 million metric tons of broiler meat [28]. Moreover, about 70–75% of the live bird weight is estimated to be the yield of poultry carcass [29]. As a result, the generation of chicken slaughterhouse wastes is high and growing, making their correct management crucial. Hence, these untapped residues must be subjected to reuse strategies in line with circular economy principles, with the aim of reducing their accumulation and pollution. Moreover, this will lead to the development of new added-value products such as fertilizers [30,31], biogas [32,33], textile materials (keratin from feathers) [34], and functional foods based on chicken proteins, hydrolysates, and peptides that exert biological activities such as the ability to reduce HTN risk [35,36].

Several chicken slaughterhouse byproducts, such as legs or feet, blood, bone residues from mechanically deboned chicken meat and cartilages, skin, feathers, trachea, viscera, and combs/wattles, may have potential as a source of antihypertensive peptides given their protein content (Table 1). In this sense, feather is a major slaughterhouse byproduct, corresponding to 5–7% of an adult chicken weight [37]. It stands out for its high protein content, reaching values up to 85% [38], with keratin being the major protein component (over 90% of total protein) [39]. Antioxidant and hypocholesterolemic effects have been observed for keratin-based peptides [40,41]. Slaughterhouses also generate blood, which corresponds to 6–7.5% of live chicken weight [42]. It is mainly intended for animal feed as it comprises 80–90% protein (dry weight) (Table 1), mainly hemoglobin (over 70% of total protein) [43]. However, antioxidant peptides have been obtained from blood [44,45]. Chicken bones come from legs, necks, backs, carcasses, etc., mainly obtained when meat is deboned. Their uses are restricted to soups, broths, and animal feed but their collagen content has placed these components as interesting materials for enzymatic hydrolysis [46,47]. Related to bones, cartilages are a source of bioactive molecules such as hyaluronic acid or chondroitin sulfate that are related to anti-atherosclerotic, anti-osteoarthritis, and antiaging effects [48]. Moreover, tracheas (or windpipes), despite their lower impact as a chicken byproduct, are very rich in proteins (69% of protein (dry weight)). Moreover, the hydrolysis of their proteins led to the release of antioxidant and antihypertensive agents [49]. Another relevant slaughterhouse byproduct is chicken skin, which corresponds to over 8–20% of chicken carcass weight together visible fat [50]. It is considered underutilized since it is a source of fatty acids, as well as collagen, elastin, or gelatin that have been hydrolyzed to obtain antioxidant and antihypertensive peptides [51,52]. Poultry slaughterhouses also generate the viscera, which includes hearts, livers, and gizzards, with high-value protein content. It is consumed by some people in different countries, but it is usually discarded or directed to aquaculture feed. Specific fractions of viscera protein hydrolysates have demonstrated free-radical-scavenging and ACEi activities [53,54,55]. Chicken feet are used in some culinary preparations, mainly in Asiatic countries; however, many countries do not consume this chicken product, and great amounts of them are usually discarded. Because of their high protein (particularly collagen) content (18–23 of protein/100 g of wet feet), they are used as a source of gelatin [56]. In addition, foot hydrolysates have been utilized as fat replacers [16,57]. Lastly, many combs and wattles, which are discarded particularly from female animals, are a source of hyaluronic acid, a potent antioxidant [58].

### 3.2. ACEi and Antihypertensive Effects of Hydrolysates from Chicken Slaughterhouse Byproducts

Chicken slaughterhouse byproducts have been used as a source of peptides able to reduce high BP. In general, these peptides were selected in vitro according to their capacity to inhibit enzymes involved in the renin–angiotensin–aldosterone system (RAAS), mainly the ACE. RAAS is an essential system involved in the regulation of cardiovascular function and fluid homeostasis, including BP regulation (see Ibarz-Blanch et al., 2022 [76]). Briefly, the system is activated when BP drops, producing the release of renin by juxtaglomerular cells. This protease cleavages the circulating angiotensinogen, releasing the peptide angiotensin (Ang) I. Then, this peptide is hydrolyzed by ACE, generating the peptide Ang II. Ang II is a key component involved in the BP increase as it produces vasoconstriction of vascular smooth muscle cells and stimulates the release of the endothelium-derived vasoconstrictor factor endothelin-1 (ET-1) and endothelial reactive oxygen species (ROS) [77,78]. Excessive ROS leads to a decrease in the production and the bioavailability of nitric oxide (NO), the main endothelial-derived vasodilator factor, which is produced by endothelial NO synthase (eNOS) action [76]. In this regard, excessive superoxide anions can scavenge NO, as well as produce “eNOS uncoupling” [79,80]. The eNOS uncoupling refers to the condition in which eNOS produces ROS (superoxide anions) instead of NO [80] (see [76] for more details about endothelial-derived factors and their relationship). Moreover, Ang II stimulates the secretion of both aldosterone by the adrenal cortex, which increases water and sodium reabsorption and potassium excretion in nephrons, and the antidiuretic hormone by the hypothalamus, which promotes the intake of water and water reabsorption in the collecting ducts of nephrons [81,82]. Thus, ACE inhibition is crucial in BP reduction not only by decreasing the Ang II production but also because it cleaves the vasodilator bradykinin, losing its vasoactive action [83].

As aforementioned, chicken byproducts are composed of a high protein content, and they have been used as a raw material for the obtainment of ACEi and antihypertensive hydrolysates and peptides.

#### 3.2.1. Blood

Several studies have been focused on the valorization of chicken blood or its components, including corpuscle and plasma fractions or isolated red blood cells, through the generation of ACEi peptides [66,84,85,86,87] (Table 2). Results from these studies indicated that ACEi activity of the blood protein hydrolysates was deeply influenced by hydrolysis conditions and protein substrate [66,84]. As expected, the hydrolytic enzyme used was one of the factors that conditioned the bioactivity of blood protein hydrolysates obtained. In that way, under the same hydrolysis conditions, Alcalase^®^ was the enzyme that generated the best ACEi hydrolysates from whole blood compared with Prozyme 6, Protease N, or Thermolysin^®^. Furthermore, a positive correlation was observed between ACEi activity and the degree of hydrolysis; a higher hydrolysis time or enzyme concentration corresponded to a higher ACEi activity [66]. Wongngam et al. (2020) also observed differences in the ACEi activity of blood-derived peptides depending on the enzyme (Alcalase^®^, Papain^®^, Pepsin, Thermolysin, and SK1-3-7), as well as on the protein substrate (whole blood, blood plasma, and blood corpuscle) employed [84]. Specifically, the most bioactive hydrolysates were those obtained by hydrolysis of blood cells with Alcalase^®^ and Thermolysin^®^, being those that also showed a higher hydrolysis degree. The optimized hydrolysis conditions with Alcalase^®^ for blood corpuscle were set to 51.1 °C for 6 h with 4% enzyme. On the other hand, the ACEi activity of this hydrolysate generated in optimum conditions increased 2.5-fold when peptides <1 kDa were obtained. Interestingly, both optimized blood corpuscle hydrolysate and its peptic fraction (<1 kDa) showed a potent antihypertensive effect in spontaneously hypertensive rats (SHR) after an acute administration. Best systolic BP (SBP) reductions were found at doses of 600 mg/kg body weight (BW) for the hydrolysate and 100–200 mg/kg BW for the fraction at 6 (57.7 mmHg) and 12 h (70.9 mmHg), respectively. The <1 kDa fraction seemed to be the best as it produced a higher and more prolonged antihypertensive effect with a lower dose than the whole hydrolysate. However, both of them showed similar BP-lowering effects in SHR administered for 4 weeks [84].

Another way to increase the ACEi activity of blood protein hydrolysates is performing the plastein reaction [86]. This effect has also been reported in the generation of soybean and casein hydrolysates [88,89]. The plastein reaction is a technique based on treating high-concentrated protein hydrolysates with proteases to form a precipitate, thixotropic colloid, or thixotropic viscous gel-like substance [90]. Peptides are released into the remaining supernatant, and it is typical to add free amino acids during the reaction [86]. In this regard, it can be noted that the ACEi activity of a hydrolysate obtained from blood plasma and trypsin (6000 U/g) at 37 °C, 5 h, and pH 8 increased through a trypsin- and papain-catalyzed plastein reaction (conditions: 15 min and 30% of hydrolysate) [86]. Moreover, a positive correlation was observed between ACEi activity and different parameters of trypsin-catalyzed plastein reaction in plasma protein including enzyme/substrate ratio (up to 6000 U/g), temperature (up to 40 °C), time (up to 5 h), and pH (up to 8). The type of exogenous amino acids added to the hydrolysate during the plastein reaction also changed the ACEi activity of the hydrolysates (leucine > tyrosine > histidine > cysteine > valine) [86]. 

Lastly, the effect of the type of hydrolysis (chemical or enzymatic) used for the generation of ACEi peptides from chicken blood proteins was also evaluated [87]. Hydrolysates obtained from isolated red blood cells using both types of hydrolysis, chemicals under acid conditions and enzymatic (Alcalase^®^), showed the same range of activity (18.7–49.4% and 14.2–47.7%, respectively). In this study, the response surface methodology was employed to optimize the hydrolysis conditions for obtaining ACEi peptides. In particular, 50 °C, 32 h, and 0.03 N HCl were established for the acid hydrolysis, whereas 60 °C, 2 h, and 2.5% Alcalase^®^ were determined for the enzymatic hydrolysis [87]. Furthermore, it was also shown that HCl concentration and temperature, and enzyme concentration for acid and enzymatic hydrolysis, respectively, were keys for the generation of hydrolysates with higher ACEi activity [87].

#### 3.2.2. Bones

Chicken bones are traditionally used as an economic source of calcium for mineral supplements; however, hydrolysates from chicken bone proteins have also shown ACEi activity (Table 2), even higher than carnosine, a dipeptide with known antihypertensive properties [91]. In this regard, it has been reported that, after 4 and 8 h of bone leg protein hydrolysis with Alcalase^®^, the obtained hydrolysates showed around 85% of ACEi activity, higher than after trypsin or pepsin hydrolysis [60]. Interestingly, the good activity in vitro of the hydrolysate was also correlated with in vivo observations, in which the 4 h hydrolysate at 50 mg/kg BW decreased the SBP of SHR after its acute administration from 2 to 8 h post administration in a similar manner to captopril at 1.5 mg/kg BW [92]. In this study, the hypotensive effect of this hydrolysate was also discarded in normotensive rats. In addition to ACEi activity, the chicken bone hydrolysate also attenuated the development of age-induced HTN and heart and vascular hypertrophy, when it was administered daily during 8 weeks [92,93]. This is of relevance as HTN was associated with the presence of cardiac hypertrophy (one of the main causes of cardiovascular mortality and morbidity), stretching of smooth muscle cells, and thickening of the vascular wall (present in several cardiovascular diseases) [94,95,96].

**Table 2:** Angiotensin-converting enzyme inhibitory and antihypertensive activities shown by the hydrolysates obtained from chicken byproducts.

By-Product		Hydrolisis Conditions	In Vitro ACEi Activity		In Vivo					References
General	Component		% *	Ic_50_ (µg/mL)	Animal Model	Oral Doses	Period	Effect on BP	Mechanism	
Blood	Corpuscle	Alcalase^®^ 4%, 6 h, 51.1 °C, pH 8.0	37.7 (0.2 mg/mL)	341	SHR	600 mg/kg BW	Single	↓ SBP (57.7 mmHg, 6 h pa)		[84]
					SHR	600 mg/kg BW	Daily for 4 weeks	↓ SBP (~60 mmHg) ↓ DBP (~30 mmHg)		
		Fraction < 1 KDa of hydrolysate (Alcalase^®^ 4%, 6 h, 51.1 °C, pH 8.0)		138	SHR	100 mg/kg BW	Single	↓ SBP (70.9 mmHg, 6 h pa) ↓ DBP (47 mmHg, 6 h pa)	-	
					SHR	100 mg/kg BW	Daily for 4 weeks	↓ SBP (~60 mmHg) ↓ DBP (~30 mmHg)	-	
	Meal	Alcalase^®^ 10%, 5 h, 50 °C, pH 8.0		340						[66]
	Isolated red blood cells	0.03 N HCl, 32 h, 50 °C	44 (ne)							[87]
		Alcalase^®^ 2.5%, 2 h, 60 °C	45 (ne)							
	Plasma	Trypsin, 6000 U/g (E/S ratio), 5 h, 37 °C, pH 7.5	54 (1 mg/mL)							[86]
Bones		Pepsin 1:100 (*w*/*w*), 6 h, 36 °C, pH 2.0		220						[97]
	Leg	Alcalase^®^ 1:50 (E/S ratio) 8 h, 50 °C, pH 8.0	86 (ne)	612 and 945						[60,92]
		Alcalase^®^ 1:50 (E/S ratio), 4 h, 50 °C, pH 8.0	84 (ne)	545 and 1960	SHR	50 mg/kg BW	Single	↓ SBP (26 mmHg, 4 h pa)		
		Alcalase^®^ 2% (E/S ratio), 4 h, 50 °C, pH 8.0		545		50 mg/kg BW	Daily, 8 weeks	Avoid increase of SBP (33 mmHg)	↑ Heart weight ↓ Heart/BW ratio ↓ Wall thickness in intramyocardial coronary vessels	[92,93]
Combs and Wattles		Alcalase^®^ 5% (E/S ratio), 4 h, 50 °C, pH 8.0		134						[98]
Feet		Protamex^®^ 0.4 AU/g prot, 2 h, 50 °C, pH 7.0		27	SHR	55 mg/kg BW	Single	↓ SBP (26.3 mmHg, 6 h pa)	↓ Plasma ACE activity	[99]
		Protamex^®^ 0.4 AU/g prot, 2 h, 50 °C, pH 7.0		9	Diet-induced hypertensive rats	55 mg/kg BW	Daily for 3 weeks	↓ SBP (~20 mmHg)	↑ GSH levels, ↓ *Et-1*, ↑ *Nox4* ↑ *Sirt1*	[100]
		Protamex^®^ 0.4 AU/g prot, 2 h, 50 °C, pH 7.0		27	SHR	85 mg/kg BW	Single	↓ SBP (30.5 mmHg, 6 h pa)		[99]
		Fraction <6000 Da: *Aspergillus oryzae* protease 0.1%		260						[101]
		Fraction <3000 Da: *Aspergillus oryzae* protease (0.1%) + Protease FP, 24 h, 50 °C, pH 7.0		130	SHR	3 g/kg wt	Single	↓ SBP (~50 mmHg, 6 h pa)		[101]
		Fraction <3000 Da: *Aspergillus oryzae* protease (0.1%) + Protease FP, 24 h, 50 °C, pH 7.0		130	SHR	3 g/kg wt	Daily for 4 weeks	↓ SBP (~33 mmHg)		
		Fraction <3000 Da: *Aspergillus oryzae* protease (0.1%) + Protease FP, 24 h, 50 °C, pH 7.0			Hypertensive rats (Wistar Kyoto rats + L-NAME)	2.0 g/kg	Single		↑ Serum NO levels (1 h pa)	[102]
		Fraction <3000 Da: *Aspergillus oryzae* protease (0.1%) + Protease FP, 24 h, 50 °C, pH 7.0			Hypertensive rats (Wistar Kyoto rats + L-NAME)	2.0 g/kg	Daily for 8 weeks	↓ SBP (~20 mmHg, 4 week pa)	↓ Hypertrophy of the arterial intima and the myofibrils in thoracic aorta ↑ Vasorelaxation of thoracic aorta ↓Plasma iCAM-1 levels	
		Fraction <3000 Da: *Aspergillus oryzae* protease (0.1%) + Protease FP, 24 h, 50 °C, pH 7.0			Mildly hypertensive subjects	5.2 g	Daily for 4 weeks	↓ SBP (11.8 mmHg, 2 weeks pa) ↓ DBP (4.1 mmHg, 2 weeks pa)	↓ Plasma renin activity (30%) ↑ EPC colonies	[103]
		Fraction <3000 Da: *Aspergillus oryzae* protease (0.1 %) + Protease FP, 24 h, 50 °C, pH 7.0			Mildly and pre-hypertensive subjects	2.9 g	Daily for 12 weeks	↓ SBP (5.3 mmHg)	↓ Brachial–ankle pulse wave velocity (righ arm and average of both arms)	[104]
Feathers		*Chryseobacterium* sp. kr6, 24 and 48 h, 30 °C, pH 8.0	53 and 65 (for 24 and 48 h, respectively) (ne)							[105]
Skins	Thigh	Alcalase^®^ 3% (*w*/*w*, protein basis), 4 h, 55 °C, pH 8.0.	80 (ne)	550	SHR	100 mg/kg BW		↓ SBP (~28–34 mmHg, 4–6 h pa)		[52,106]
	Breast	Pepsin 1% (*w*/*w*, protein basis), 2 h, 37 °C, pH 2.0 + Pancreatin 1% (*w*/*w*, protein basis), 4 h, 37 °C, pH 7.5	~78 (ne)	640	SHR			↓ SBP (31 mmHg, 6 h pa)		
	Thigh and breast	Mixture of two hydrolysates (1:1) Thigh + Alcalase^®^ 3% (*w*/*w*, protein basis), 4 h, 55 °C, pH 8.0; Breast + pepsin 1% (*w*/*w*, protein basis), 2 h, 37 °C, pH 2.0 + Pancreatin 1% (*w*/*w*, protein basis), 4 h, 37 °C, pH 7.5				0.5%	Daily for 6 weeks	↓ SBP (31 mmHg)	↓ Plasma ACE activity	[107]
	Thigh and breast	Mixture of two hydrolysates (1:1) Thigh + Alcalase^®^ 3% (*w*/*w*, protein basis), 4 h, 55 °C, pH 8.0; Breast + pepsin 1% (*w*/*w*, protein basis), 2 h, 37 °C, pH 2.0 + Pancreatin 1% (*w*/*w*, protein basis), 4 h, 37 °C, pH 7.5			SHR	1%	Daily for 6 weeks	↓ SBP (36 mmHg)	↓ Plasma ACE activity ↑ Urine creatinine ↑ Urine L-isoleucine ↓ Urine uric acid ↓ Urine N_2_-acetyl-ornithine ↑ Urine N_1_-acetylspermidine ↓ Urine symmetric dimethylarginine ↑ Urine pentahomomethionine ↓ Urine buthionine sulfoximine ↓ Plasma tranexamic acid ↑ Plasma 13-docosenamide ↓ Plasma Vitamin E succinate ↑ Plasma PS(O-16:0/15:0) ↑ Plasma PS(O-18:0/15:0)	
Trachea		Alcalase^®^ 1% (*w*/*w* protein), 1 h, 50 °C, pH 8.0		422						[49]
Residues	Mixture	Alcalase^®^ 1%, (*w*/*w* residue), 2.5 h, 60 °C		273	SHR	3%	Daily for 16 weeks	↓ SBP (26 mmHg)	↓ Aorta ACE activity	[108]
Viscera	Intestine, spleen, gall bladder, and connective tissues	Autolytic degradation of tissue protein (6 h, 55 °C, pH 2.8)		350–2650						[54,55]
	Liver	*Pediococcus acidilactici*N-CIM5368 24 h, 37 °C pH 4.0 or Alcalase^®^ 2.5 L, 1.5% (*v*/*w*), 1.5 h, 45 °C			Cyclophosphamide-induced anemic mice (Swiss-albino female mice)	Diet deficient in iron + 1.5%, 3%, and 4.5%	Daily for 4 weeks	Restore BW Restore hemoglobin levels ↑ Plasma antioxidant activity		[109]
		Alcalase^®^ 2.4 L and Flavourzyme^®^ 500 L (1:1), 2 h, 50 °C	81 (ne)							[53]

Abbreviations: ACEi activity: angiotensin-converting enzyme-inhibitory activity; BP: blood pressure; BW: body weight; DBP: diastolic blood pressure; EPC: endothelial progenitor cells; *Et-1*; endothelin 1 gene; E/S: enzyme/substrate; GSH: reduced glutathione; L-NAME: N(ω)-nitro-L-arginine methyl ester; ne: not specified; NO: nitric oxide; *Nox4*: NADPH oxidase subunit 4 gene; pa: post administration; PS; phospholipids; SBP: systolic blood pressure; SHR: spontaneously hypertensive rats; *Sirt1*: sirtuin 1 gene; * Numbers in brackets indicate the concentration of protein for testing the ACEi activity. ↑ and ↓ indicate increase or decrease of a parameter, respectively.

#### 3.2.3. Chicken Feet/Legs/Claws

Chicken feet, also known as legs or claws (yellow part of the legs), have been used to obtain ACEi hydrolysates (Table 2). In particular, the first study using this byproduct for this purpose was conducted in 2008 [101] and showed good ACEi activity in the fraction <6000 Da of a chicken-leg protein hydrolysate obtained with *Aspergillus oryzae* proteases (0.1%). Moreover, its bioactivity was increased when it was further processed with other enzymes (protease FP, protease N, or protease A amano G) or when it was subjected to a gastrointestinal digestion process using 1% pepsin and trypsin/chymotrypsin at 37 °C and pH 7.0 for 1 h [101]. In addition to ACEi, the fraction <3000 Da of the hydrolysate obtained with *Aspergillus oryzae* proteases and further hydrolyzed with the FP protease also showed antihypertensive effects in SHR after an acute and a long-term administration [101]. Moreover, the mechanisms involved in this antihypertensive effect were also investigated in N(ω)-nitro-L-arginine methyl ester (L-NAME)-induced hypertensive rats [102]. Concretely, animals administered with the hydrolysate showed higher serum NO levels, better aorta vasodilatation, a lower grade of aorta hypertrophy, and lower plasma intercellular adhesion molecule-1 (iCAM-1) levels than the hypertensive control group. These results reflected an improvement in the cardiovascular system, including in the endothelial functionality. In this regard, the endothelium also plays a key role in BP regulation by producing molecules with vasoactive actions, holding blood fluidity, and regulating vascular tone, vascular smooth muscle cell functionality, and immune and inflammation response [110,111]. Endothelial dysfunction has been associated with different diseases including HTN, atherosclerosis, and diabetes [112]. It is characterized by an imbalance in the proportion of endothelial-derived vasodilator (NO, prostaglandin) and vasoconstrictor (ET-1) factors, a reduction in the bioavailability of the endothelial vasodilator NO, and an increase in the vascular smooth muscle contractibility [113,114]. In that way, it is important to mention that serum levels of iCAM-1 have been associated with endothelial dysfunction and other CVD such as myocardial infarction, coronary heart disease, carotid, and some types of atherosclerosis [115,116,117,118]. iCAM-1, whose expression is upregulated by cytokines in endothelial cells, is involved in the formation and development of atherosclerotic lesions as it stimulates the adhesion of leukocytes to the endothelium and their transmigration into the subendothelial space [119]. On the other hand, the BP-lowering effects of this leg-derived hydrolysate were also validated in prehypertensive and mildly hypertensive subjects. In this regard, a significant reduction in SBP was observed in these volunteers after consuming 5.2 g/day of the hydrolysate for 2 weeks. Its effects were associated with its action on the RAAS and vascular endothelium as a strong reduction in the plasma renin activity and a significant increase in the blood endothelial progenitor cells were observed in these treated volunteers [103]. Endothelial progenitor cells are involved in vascular repair [120]. Moreover, a significant reduction in SBP was also reported after consumption of 2.9 g/day for mildly and prehypertensive subjects with respect to control volunteers [104]. The ingestion of the hydrolysate also led to a reduction in the brachial–ankle pulse wave velocity. This parameter indicates arterial stiffness and is a potential predictor of HTN and stroke development [121].

More recently, our group obtained a chicken foot hydrolysate denominated Hpp11, characterized by an excellent ACEi activity (IC_50_ = 27 µg/mL). The enzymatic solution Protamex^®^ was employed for the generation of this hydrolysate [99]. A considerable reduction in SBP was shown after acute administration at 55 and 85 mg/kg BW. Specifically, the maximum effect was observed at 6 h post administration (−26.33 and −30.45 mmHg for 55 and 85 mg/kg BW, respectively). The BP-lowering effects were similar to those found with a high dose of the antihypertensive drug captopril (50 mg/kg BW) [99]. An undesirable hypotensive effect was also discarded in normotensive rats. Moreover, it is important to highlight that Hpp11 (55 mg/kg BW) showed functionality after a long-term administration (3 weeks), observing BP-lowering effects from the first week of consumption until the end of the study [100]. Its impact on BP in diet-induced hypertensive rats was associated with an improvement in both the oxidative stress, increasing hepatic reduced glutathione (GSH) levels, and in the endothelial dysfunction, downregulating *Et-1* expression and upregulating *Sirt-1* expression in aorta [100]. The effects of sirtuin 1 (SIRT-1) on the endothelium are linked to a vasodilator function, acting on endothelial NO production and bioavailability. Specifically, it activates eNOS by deacetylation, stimulates the transcription of *eNos*, and inhibits the activity of NADPH oxidase (NOX), reducing the production of ROS by NOX [76,122,123]. Lastly, Hpp11 was combined with other bioactive compounds including a grape seed proanthocyanidin extract, a berry anthocyanidin extract, and conjugated linoleic acid in order to elaborate a multifunctional product to manage metabolic syndrome [124,125,126]. This product also exerted a potent reduction in SBP in diet-induced hypertensive rats after its daily intake for 3 weeks [125].

#### 3.2.4. Skins

The ACEi activity of skin hydrolysates is influenced by the enzyme type and concentration used in the hydrolysis process, as well as by the skin type. Thus, hydrolysis using Alcalase^®^ produced hydrolysates with better activity than the combination of pepsin/trypsin in skin from both breast and thigh [52]. Hydrolysates from both skin breast proteins with 1% pepsin/pancreatin and skin thigh proteins with 3% Alcalase^®^ also showed a potent antihypertensive effect that lasted until 24 h post administration [106]. When the combination of both hydrolysates was administered at 0.5% and 1% BW to SHR for 6 weeks, relevant antihypertensive effects were observed from the first week of administration, reaching a plateau at the third week of treatment. A metabolomics study indicated that this mixture of skin hydrolysates acted on the RAAS system and vascular function [107]. Additionally, a reduction in plasma ACE activity was observed, together with an increase in urine creatinine levels, which was associated with the arginine metabolism and NO production (Table 2). In contrast, a decrease in urine uric acid and plasma α-tocopherol succinate (vitamin E succinate) was also observed, which would be indicative of a reduction in oxidative stress [107]. Elevated serum uric acid levels have been related to the development of HTN as it produces oxidative stress, which would reduce the endothelial NO availability, and acts on RAAS system, increasing both intrakidney Ang activity and plasma renin activity [127]. It is known that α-tocoferol, a potent antioxidant, can modulate the activation and or transcription of several genes involved in oxidative stress including NOX, superoxide dismutase, NOS, or ciclooxigenase-2, leading to its reduction [128]. Thus, a reduction in urine α-tocoferol succinate would be indicative of increased free-radical scavenging [107]. Sarbon and colleagues also demonstrated the antihypertensive effects of peptides isolated from gelatin from chicken skin hydrolyzed with Collagenase, Alcalase^®^, and Pronase E. After ultrafiltration, the isolation of small-molecular-weight peptides increased the ACEi activity of the hydrolysates, which was comparable to that obtained with captopril [129]. In the in vivo study, a single administration of the peptides was more effective than losartan in reducing the SBP of SHR for 24 h.

#### 3.2.5. Other Chicken Byproducts

Although much less studied, feather, trachea, viscera, combs, wattles, and chicken residues (unspecified residues) have also been used to obtain peptides with in vitro ACEi activity (Table 2) [49,53,98,105,108]. However, the antihypertensive effects of these hydrolysates remain unexplored. In this regard, two independent studies showed that Alcalase^®^ was the best enzyme in producing ACEi peptides using trachea and chicken residues in comparison to the enzyme solutions Flavourzyme^®^, Protamex^®^, Papain^®^, Bromelain^®^, or Esperase^®^ [49,108]. However, the best results in viscera were found when it was treated with Neutrase^®^ 0.8 L, a binary mixture of Flavourzyme^®^ 500 L and Alcalase^®^ 2.4 L (1:1), and a ternary mixture of Flavourzyme^®^ 500 L, Alcalase^®^ 2.4 L, and Neutrase^®^ 0.8 L [53]. Regarding feathers, they were fermented with *Chryseobacterium* sp. kr6 [105], a bacterium isolated directly from feathers, which produces four different extracellular alkaline keratinases [91]. This process helps to solubilize feather proteins, producing in turn bioactive peptides [105].

### 3.3. ACEi and Antihypertensive Effects of Chicken Byproduct-Derived Peptides

In addition to the ACEi activity and antihypertensive effects of the hydrolysates, some studies have identified the peptides involved in the bioactivity of the hydrolysates (Table 3). Generally, the amino-acid composition of the peptides, especially at both ends of the sequence, determines their ACEi activity. In this regard, it has been observed that the presence of hydrophobic residues such as Trp, Tyr, Pro, or Phe at the C-terminal end, as well as aliphatic amino acids such as Gly, Val, Leu, and Ile at the N-terminal position, leads to higher ACE inhibitions [130,131,132]. Specifically, ACEi peptides have been identified in hydrolysates from feet, blood corpuscle, legs with claws, bone, viscera, or a mixture of combs and wattles, showing IC_50_ values lower than 100 µM in many cases [22,85,97,98,133]. The protein source of these peptides depends on the used byproduct. For example, in combs and wattles, the main sources of ACEi peptides were collagen and elastin [98], while, in blood corpuscle, the main sources were cytochrome Bc1, hemoglobin, and fibrinogen, among others [85]. In addition, antihypertensive effects of peptides isolated from feet, bone, and blood corpuscle hydrolysates have also been evidenced in SHR [22,97,101,134]. Concretely, these were the amino-acid sequences QVGPLIGRYCG, AVFQHNCQE, VSKRLNGDA, GAOGLOGP, and YYRA (Table 3), which had a length between four and 11 amino-acid residues. In addition, these peptides showed an excellent ACEi activity, which could be associated to the presence of Val and/or hydrophobic amino acids/Glu at their N- and C-terminal ends, respectively [22,23]. In this regard, the peptides QVGPLIGRYCG and AVFQHNCQE, isolated from a Protamex^®^-digested foot hydrolysate, showed antihypertensive effects in SHR (10 mg/kg BW), exerting the maximum BP reduction at 6 h post administration (−10.94 and −25.07 mmHg, respectively) [22]. Additional studies showed that AVFQHNCQE, which is hydrolyzed during gastrointestinal digestion and seems not to be absorbed by gastrointestinal tract, displays an antihypertensive action through an increase in NO levels, which is mediated by the activation of opioid receptors [135]. After the administration of this peptide, SHR also showed an improvement in endothelial dysfunction and a reduction in oxidative stress markers with respect to water controls [136]. Another example of a known bioactive peptide is the amino-acid sequence GAOGLOGP, isolated from a leg hydrolysate, which also produced a potent reduction of BP in SHR, reaching the maximum at 6 h post administration [134]. In vitro studies carried out in Caco-2 cells, which are used to simulate the intestinal barrier, showed the energy-independent paracellular transport of the intact peptide [137]. In addition, this peptide produced the release of NO and the activation of eNOS through its phosphorylation at Ser^1179^ when it was added to bovine aortic endothelial cells, suggesting the potential effect of this peptide in the vascular function [137]. Lastly, VSKRLNGDA is another peptide that has been widely studied to elucidate the mechanisms involved in its antihypertensive effects. This peptide, identified in a blood corpuscle hydrolysate, reduced both SBP and DBP in SHR after its acute administration at 12.5, 25, and 50 mg/kg BW [85]. Interestingly, at 12 h post administration, the dose of 50 mg/kg BW continued exerting its antihypertensive effect, especially noted in SBP (41.8 mmHg). After a prolonged administration to SHR (4 weeks), this peptide at 50 mg/kg BW also showed a potent antihypertensive effect, which was associated with its action on different RAAS system targets, e.g., downregulating the genes encoding renin (*Ren1*) and Ang-II type-1 receptor (*Agtr1b*) in kidney. Thus, the peptide would reduce the Ang-II effects on HTN acting in the Ang II-production pathway and the Ang-II receptor involved in the Ang-II effects associated with a BP increase. In addition, VSKRLNGDA also upregulated the expression of adrenoceptor β-3 (*Adrb3*) and peroxisome proliferator-activated receptor δ (*Ppard*) in kidneys of treated SHR [85], thus contributing to a BP reduction. Specifically, adrenoceptor β-3 activation has been coupled to an increase in NO levels via eNOS activation [138], and *Ppard* activation has been associated with a reduction in the proatherogenic, proinflammatory, and oxidative state of hypertensive rats, as well as a restoration of the vascular function and structure [139]. In contrast to the ACEi effect of the five antihypertensive amino-acid sequences isolated from chicken byproduct hydrolysates, the BP-lowering effects of these peptides could not be related to a specific amino-acid profile. Thus, their in vivo effects may be a consequence of the peptide-derived fragments released during the gastrointestinal enzymatic digestion of the chicken peptides, as seen for the amino-acid sequences AVFQHNCQE or VSKRLNGDA [85,135].

## 4. Conclusions

Chicken slaughterhouse byproducts are a valuable source of ACEi activity and antihypertensive effects. Among these byproducts, chicken feet hydrolysates have been the most studied, showing BP-lowering effects in both animal and human studies. Some of these hydrolysates also exerted antioxidant effects, improved HTN-associated endothelial dysfunction, and reduced ACE activity in different in vivo hypertensive models, which may contribute to their antihypertensive activity. Although some peptides have been identified in these hydrolysates, few studies have validated their in vivo effects.

Taken altogether, chicken byproducts may be excellent candidates for the management of HTN. However, further investigations in clinical studies are needed in order to confirm their beneficial effects observed in hypertensive animal models. This would lead to the possibility to increase the value of these food industry wastes contributing also to the circular economy model of slaughterhouses.

## Figures and Tables

**Table 1:** Composition of chicken byproducts (% wet basis).

Chicken By-Products	Protein (%)	Ash (%)	Moisture (%)	Fat (%)	Carbohydrate (%)	Mineral (%)	Fiber (%)	References
Bones	15.6–23.58	11.80–12.35	53.2–57.51	8.4–9.52	1	14.7–15.9	-	[46,59,60,61]
Feathers	80–85.31	0.69–0.83	2.03–10.06	2–3.92	-	1.2	<1	[38,62,63,64]
Blood	14.46–20.55	0.81–3.74	75–82	0.033–1.69	0.363	-	-	[43,65,66]
Skin	8.5–15.21	0.64–0.9	43.70–54.22	31.44–41.4	-	-	-	[67,68,69]
Feet/legs	18.10–22.91	7.94–8.16	58.02–65.08	3.90–6.2	-	-	-	[70,71,72]
Viscera	11.2–12.8	1.1–1.76	69.64–76.5	6.9–16.93	-	-	-	[73,74]
Trachea *	69.71	-	-	16.53	-	-	-	[49]
Crest	-	-	84	-	-	-	-	[58]
Cartilages	11.78	1.21	82.85	0.29	3.87	-	-	[75]

* % (g/100 g dry byproduct).

**Table 3:** Angiotensin-converting enzyme-inhibitory and antihypertensive activities shown by the peptides identified in hydrolysates obtained from chicken byproducts.

By-Product	Hydrolisis Conditions	Amino Acid Sequence	Native Protein	ACEi Activity (µM)	Model	Dose (mg/kg BW)	Period	Effect on BP	Mechanism	Reference
Feet	Protamex^®^ 0.4 AU/g prot, 2 h, 50 °C, pH 7.0	LGIHPDWQFV	ne	>137.6						[22]
		LSETVV	ne	>515.4						
		LSGPVKF	ne	80.9						
		AVKILP	ne	7.1						
		VRWEPAPGPV	ne	>150.0						
		VGKPGARAPmY	ne	29.7						
		QVGPLIGRYCG	ne	11.0	SHR	10	Single	↓ SBP (10.9 mmHg, 6 h pa)		
		AVFQHNCQE	ne	44.8	SHR	10	Single	↓ SBP (25.1 mmHg, 6 h pa) ↓ DBP (17.7 mmHg, 2 h pa)	Implication of NO ↓ *Nox4* ↓ *Et-1* ↑ GSH levelsActivation opiod receptors	[22,135,136]
Blood corpuscle	Alcalase^®^ 4% enzyme, 6 h, 51.1 °C, pH 8.0	VNEDSGPFEDSTGATS	Chain I, cytochrome Bc1 complex from chicken with designed inhibitor bound	35.56						[85]
		VSKRLNGDA	Chain B, R-state form of chicken hemoglobin D	34.48	SHR	12.5, 25 and 50	Single	↓ SBP ↓ DBP		
					SHR	50	Daily for 4 weeks	↓ SBP (83.1 mmHg) ↓ DBP	*↓ Ren1* *↓ Agtr1b* *↑ Adrb3* *↑ Ppard*	
		MMTCLAGMPNLF	Chain C, cytochrome Bc1 complex from chicken	40.64						
		ELNNLLNPALFFSA	Chain D, chicken cytochrome Bc1 complex inhibited by an iodinated analog of the polyketide crocacin-d	34.48						
		ARCGSHCDYIKHWP	Chain B, chicken cytochrome Bc1 complex inhibited by an iodinated analog of the polyketide crocacin-d	29.17						
		NVSTVLTMKKF	Chain A, R-state form of chicken hemoglobin D	40.64						
		CSFDVPTGWASWTPL	Chain A, two fibronectin type iii domain segment from chicken tenascin	39.24						
		FPLCTPAFMTV	Chain I, cytochrome Bc1 complex from chicken	29.17						
		NCVWSGSTFGNPRYSIG	Chain A, crystal structure of native chicken fibrinogen	31.60						
		VMKKSSRCTGFERLAGFNRNFEFA	Chain G, chicken cytochrome Bc1 complex inhibited by an iodinated analog of the polyketide crocacin-d	51.73						
Leg with claws	*Aspergillus oryzae* protease (0.1%) + Protease FP, 24 h, 50 °C, pH 7.0	GAOGLOGP	Collagen α1	29.4	SHR	4.5 mg/kg BW	Single	↓SBP (~38 mmHg, 6 h pa)		[101,133]
					Bovine aortic endothelial cells (BAECs)	10 µM		↑ NO levels ↑ Phosphorylation of eNOS at Ser^1179^		[137]
		GAOGPAGPGGIOGERG	Collagen α2	45.6						[101]
		GLOGSRGERGLOG	Collagen α2	60.8						
		GIOGERGPVGPSG	Collagen α2	43.4						
Bone	Pepsin 1:100 (*w*/*w*), 6 h, 36 °C, pH 2.0	YYRA	g heavy chain V region	33.9	SHR	10 mg/kg	Single	↓ SBP (~18 mmH, 3–6 h pa)		[97]
Combs and Wattles	Alcalase 5% (E/S ratio), 4 h, 50 °C, pH 8.0	APGLPGPR		53						[98]
		Piro-GPPGPT		88						
		FPGPPGP		38						
Viscera	Autolytic degradation of tissue protein (6 h, 58 °C, pH 2.8)	ARIYH	Peripheral myelin protein 22a	13.6						[55]
		LRKGNLE	Basic leucine zipper and W2 domain-containing protein 2	10.8 ± 0.5						
		RVWCP	NADH dehydrogenase [ubiquinone] 1 alpha subcomplex assembly factor 4 isoform X2	7.5 ± 0.3						

Abbreviations: ACEi activity: angiotensin-converting enzyme inhibitory activity; *Adrb3*: adrenoceptor beta 3 gene; *Agtr1b*: angiotensin II receptor type 1 b gene; BP: blood pressure; BW: body weight; DBP: diastolic blood pressure; eNOS: endothelial nitric oxide synthase; *Et-1*: endothelin 1 gene; E/S: enzyme/substrate; GSH: reduced glutathione; ne: not specified; NO: nitric oxide; *Nox4*: NADPH oxidase subunit 4 gene; pa: post administration; *Ppard*: peroxisome proliferator-activated receptor delta gene; SBP: systolic blood pressure; SHR: spontaneously hypertensive rats; *Ren1*: renin gene. ↑ and ↓ indicate increase or decrease of a parameter, respectively.

## Data Availability

Not applicable.
